# Supplementary material for: Rank-Reduced Equation-of-Motion Coupled Cluster Triples: an Accurate and Affordable Way of Calculating Electronic Excitation Energies
Source: J Chem Theory Comput. 2024 Sep 30;20(20):8970–83. doi: 10.1021/acs.jctc.4c00959 (PMC11500429; doi:10.1021/acs.jctc.4c00959)
Supplement: Supplementary file 1 — ct4c00959_si_001.pdf [file ct4c00959_si_001.pdf]

Supporting Information for the paper: Rank-reduced equation-of-motion coupled cluster triples: an accurate and affordable way of calculating electronic excitation energies

Piotr Michalak and Michał Lesiuk*

Faculty of Chemistry, University of Warsaw, Pasteura 1, Warsaw, 02-093, Poland

E-mail: m.lesiuk@uw.edu.pl

Contents

1 Impact of the triple-excitation subspace size 2

2 Molecular structures 3

2.1 Small molecules 4

2.2 Molecules with doubly-excited states[1] 5

2.3 Larger molecules 7

References 8

1 Impact of the triple-excitation subspace size

Here we discuss how the choice of the triple-excitation subspace sizes for the ground-state (N_{svd}) and the excited state (N_{SVD}) impacts the accuracy of the RR-EOM-CC3 method. The results are presented in Fig. S1 in a form of two heatmaps (for aug-cc-pVDZ and aug-cc-pVTZ basis sets). The heatmaps show the absolute value of the difference in the first excitation energies calculated with the RR-EOM-CC3 method and the exact EOM-CC3, plotted against N_{svd} and N_{SVD} parameters, and averaged over the chosen set of molecules (BH_3, C_2H_2, C_2H_4, CH_3OH, CO, H_2O, CH_2O, NH_3). In order to produce the heatmaps we calculated the errors for a grid of 225 points representing different combinations of N_{SVD} (Y axis) and N_{svd} (X axis) values. The investigated subspace sizes for each molecule were in turn calculated according to the following formulas:

$$N_{\mathrm{SVD}} = N_{\mathrm{MO}} \cdot y, \tag{1}$$

$$N_{\mathrm{svd}} = N_{\mathrm{MO}} \cdot x, \tag{2}$$

where x and y are taken from a set of 15 equidistant points $\{0.500, 0.571, 0.642, \ldots, 1.494\}$ and N_{MO} is a number of molecular orbitals of a given molecule (excluding frozen-core orbitals). The obtained subspace sizes were then rounded to the nearest integer.

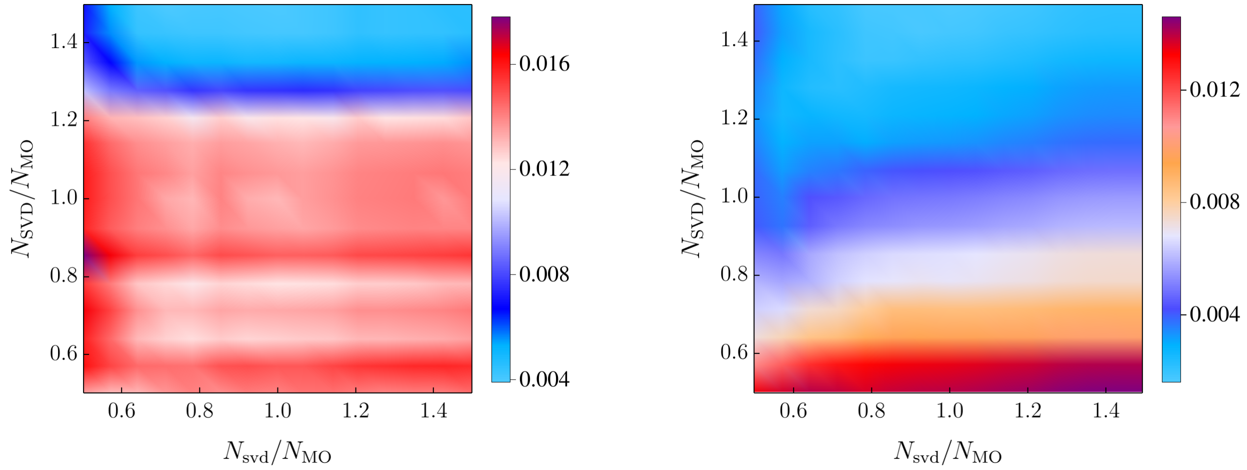

Figure S1: Comparison of the RR-EOM-CC3 with the exact EOM-CC3 in aug-cc-pVDZ (left panel) and aug-cc-pVTZ (right panel) basis sets. Colors indicate the absolute difference, in eV, between the first excitation energies calculated with both methods. The error is plotted as a function of the sizes of triple excitation subspaces for the ground state (N_{svd}) and the excited state (N_{SVD}) divided by the number of molecular orbitals of a given molecule (N_{MO}). The presented results are averaged over the chosen set of systems.

In the investigated range of parameters we observe medium to small errors in both basis sets as compared to the mean absolute value of the error of the exact EOM-CC3 which equals 0.03 eV for states of singly-excited character (in comparison to the FCI results).[2] The errors for aug-cc-pVDZ basis set range from approximately 0.004 to 0.018 eV, while for aug-cc-pVTZ basis set they are between roughly 0.002-0.015 eV. We note that the error seems to be more strongly dependent on the triple-excitation subspace size for the excited state than for the ground state. For a given N_{SVD}, expanding the ground-state parameter N_{svd} often do not change the result significantly. On the other hand, expansion of N_{SVD} in general improves the accuracy of the obtained excitation energy.

2 Molecular structures

Here we provide the optimized geometries (Cartesian coordinates) for all molecules studied in the present work. They are either in angstrem (small molecules and L-proline) or bohr (doubly-excited states and heptazine). In the case of small molecules the geometries were

calculated at the B3LYP-D3/cc-pVTZ level in PSI4 quantum chemistry program. The rest of the geometries were taken from the literature, as cited below. We include them for reader's convenience.

2.1 Small molecules

BH_3 molecule

B	0.000000000000	0.000000000000	−0.000000074111
H	0.000000000000	0.000000000000	1.188614756918
H	1.029372683910	0.000000000000	−0.594306973673
H	−1.029372683910	0.000000000000	−0.594306973673

C_2H_2 molecule

C	0.000000000000	0.000000000000	0.598118722493
C	0.000000000000	0.000000000000	−0.598118722493
H	0.000000000000	0.000000000000	1.659924032560
H	0.000000000000	0.000000000000	−1.659924032560

C_2H_4 molecule

C	0.000000000000	0.000000000000	0.662219700123
C	0.000000000000	0.000000000000	−0.662219700123
H	0.000000000000	0.920809943487	1.231848156585
H	0.000000000000	−0.920809943487	1.231848156585
H	0.000000000000	0.920809943487	−1.231848156585
H	0.000000000000	−0.920809943487	−1.231848156585

CH_3OH molecule

C	−0.026074566367	0.728168754206	0.000000000000
O	−0.026444431728	−0.693594510308	0.000000000000
H	−1.068225463593	1.043851747083	0.000000000000
H	0.456931811866	1.146393372142	0.890095357408
H	0.456931811866	1.146393372142	−0.890095357408
H	0.884519556251	−0.998970893166	0.000000000000

CO molecule

C	0.000000000000	0.000000000000	−0.643436329748
O	0.000000000000	0.000000000000	0.482730676632

H_2O molecule

O	0.000000000000	0.000000000000	0.065831956999
H	0.000000000000	0.760367790528	−0.522400465245
H	0.000000000000	−0.760367790528	−0.522400465245

CH_2O molecule

O	0.000000000000	0.000000000000	0.599739499023
C	0.000000000000	0.000000000000	−0.599705973764
H	0.000000000000	0.937371727823	−1.188852389581
H	0.000000000000	−0.937371727823	−1.188852389581

NH_3 molecule

N	−0.000000298243	−0.068242713259	0.000000000000
H	0.938149899945	0.316069874921	0.000000000000
H	−0.469072878024	0.316059143096	0.812462152836
H	−0.469072878024	0.316059143096	−0.812462152836

2.2 Molecules with doubly-excited states[1]

Acrolein

C	−1.11645072	−0.68348783	0.00000000
C	1.20647847	0.83714564	0.00000000
C	3.46831059	−0.28872636	0.00000000
O	−3.23666415	0.19187203	0.00000000
H	−0.80613858	−2.74747338	0.00000000
H	0.98699813	2.86613511	0.00000000
H	5.20930864	0.77443560	0.00000000
H	3.60951559	−2.33000749	0.00000000

Butadiene

C	1.14656244	0.00000000	0.75468820
C	−1.14656244	0.00000000	−0.75468820
C	3.48132647	0.00000000	−0.22482805
C	−3.48132647	0.00000000	0.22482805
H	0.90770978	0.00000000	2.78883925
H	−0.90770978	0.00000000	−2.78883925
H	3.77525814	0.00000000	−2.24895470
H	−3.77525814	0.00000000	2.24895470
H	5.13664967	0.00000000	0.96861890
H	−5.13664967	0.00000000	−0.96861890

Benzene

C	0.00000000	2.63144965	0.00000000
C	−2.27890225	1.31572483	0.00000000
C	−2.27890225	−1.31572483	0.00000000
C	0.00000000	−2.63144965	0.00000000
C	2.27890225	−1.31572483	0.00000000
C	2.27890225	1.31572483	0.00000000
H	−4.04725813	2.33668557	0.00000000
H	−4.04725813	−2.33668557	0.00000000
H	0.00000000	−4.67337115	0.00000000
H	4.04725813	−2.33668557	0.00000000
H	4.04725813	2.33668557	0.00000000
H	0.00000000	4.67337115	0.00000000

Nitrosomethane

C	−1.78426612	0.00000000	−1.07224050
N	−0.00541753	0.00000000	1.08060391
O	2.18814985	0.00000000	0.43452135
H	−0.77343975	0.00000000	−2.86415606
H	−2.97471478	1.66801808	−0.86424584
H	−2.97471478	−1.66801808	−0.86424584

Nitroxyl

O	0.21099695	0.00000000	2.15462460
N	−0.44776863	0.00000000	−0.03589263
H	1.18163475	0.00000000	−1.17386890

Glyoxal

C	1.21360282	0.75840215	0.00000000
C	−1.21360282	−0.75840215	0.00000000
O	3.25581408	−0.26453186	0.00000000
O	−3.25581408	0.26453186	0.00000000
H	0.96135276	2.81883243	0.00000000
H	−0.96135276	−2.81883243	0.00000000

2.3 Larger molecules

L-proline[3,4]

O	2.1617	−0.9509	−0.1119
O	1.5688	1.2164	−0.4953
N	−0.7807	0.9314	0.7770
C	0.0834	−0.2476	0.7251
C	−0.7235	−1.3171	−0.0031
C	−1.9888	−0.6106	−0.4752
C	−1.6499	0.8635	−0.3933
C	1.3289	0.1149	−0.0235
H	0.3625	−0.5522	1.7389
H	−0.9873	−2.1429	0.6671
H	−0.1919	−1.7411	−0.8624
H	−2.2798	−0.9157	−1.4848
H	−2.8187	−0.8485	0.2018
H	−1.1204	1.1932	−1.2945
H	−2.5424	1.4831	−0.2694
H	−0.2509	1.8003	0.8023
H	2.9837	−0.7341	−0.6014

Heptazine[5]

C	0.00000000	−2.28707566	1.32044375
C	0.00000000	−4.25961422	−2.45928942
C	0.00000000	0.00000000	−2.64088750
C	0.00000000	4.25961422	−2.45928942
C	0.00000000	2.28707566	1.32044375
C	0.00000000	0.00000000	4.91857884
N	0.00000000	0.00000000	0.00000000
N	0.00000000	−4.45372767	0.04447145
N	0.00000000	2.18835043	−3.87927703
N	0.00000000	2.26537724	3.83480558
N	0.00000000	−2.26537724	3.83480558
N	0.00000000	−2.18835043	−3.87927703
N	0.00000000	4.45372767	0.04447145
H	0.00000000	6.02612802	−3.47918663
H	0.00000000	0.00000000	6.95837327
H	0.00000000	−6.02612802	−3.47918663

References

(1) Loos, P.-F.; Lipparini, F.; Matthews, D. A.; Blondel, A.; Jacquemin, D. A Mountaineering Strategy to Excited States: Revising Reference Values with EOM-CC4. *J. Chem. Theory Comput.* **2022**, *18*, 4418–4427.

(2) Loos, P.-F.; Scemama, A.; Blondel, A.; Garniron, Y.; Caffarel, M.; Jacquemin, D. A mountaineering strategy to excited states: Highly accurate reference energies and benchmarks. *J. Chem. Theory Comput.* **2018**, *14*, 4360–4379.

(3) Paul, A. C.; Myhre, R. H.; Koch, H. New and Efficient Implementation of CC3. *J. Chem. Theory Comput.* **2021**, *17*, 117–126.

(4) National Center for Biotechnology Information. PubChem Compound Summary for CID 145742, Proline. https://pubchem.ncbi.nlm.nih.gov/compound/Proline, Accessed July 14, 2024.

(5) Loos, P.-F.; Lipparini, F.; Jacquemin, D. Heptazine, Cyclazine, and Related Compounds:

Chemically-Accurate Estimates of the Inverted Singlet–Triplet Gap. *J. Phys. Chem. Lett.* **2023**, *14*, 11069–11075.
